# Supplementary material for: An mRNA processing pathway suppresses metastasis by governing translational control from the nucleus
Source: Nat Cell Biol. 2023 May 8;25(6):892–903. doi: 10.1038/s41556-023-01141-9 (PMC10264242; doi:10.1038/s41556-023-01141-9)
Supplement: Supplementary file 1 — Supplementary Figure exemplifying the gating strategy for FACS. [file 41556_2023_1141_MOESM1_ESM.pdf]

nature cell biology

Article https://doi.org/10.1038/s41556-023-01141-9

An mRNA processing pathway suppresses metastasis by governing translational control from the nucleus

In the format provided by the authors and unedited

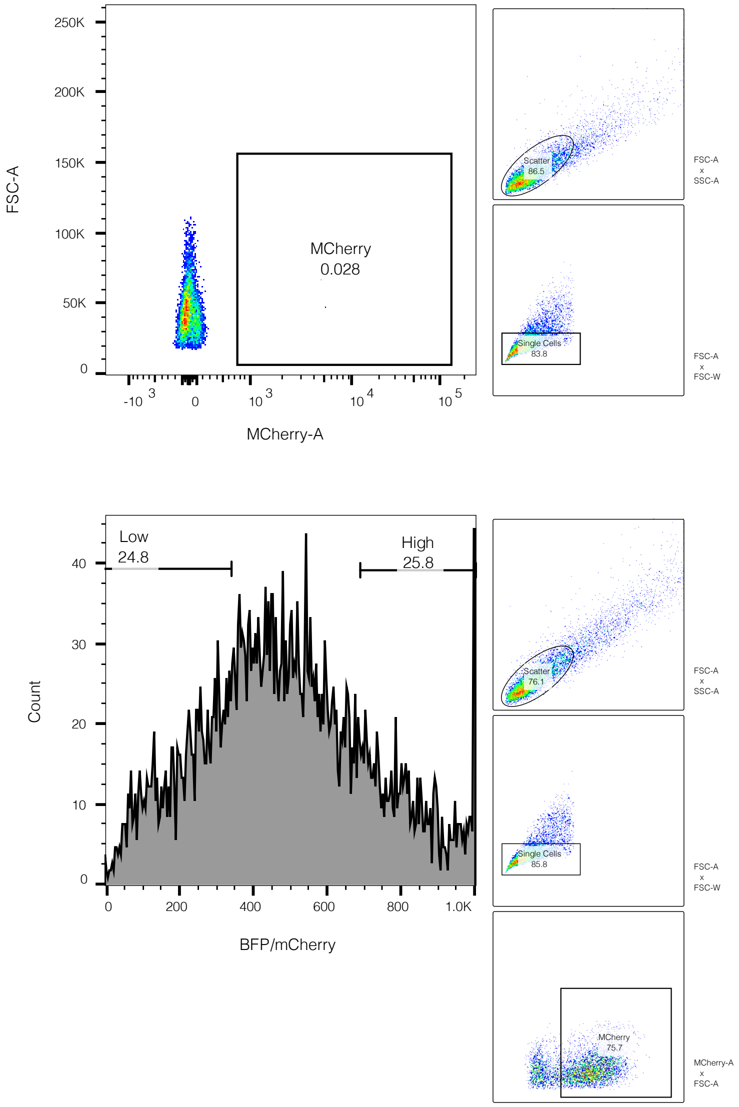

Gating strategy for flow cytometry and cell sorting experiments. Untransduced (top panel) or reporter transduced (bottom panel) MDA-MB-231 cells were gated based on forward and side scatter area (FSC-A x SSC-A), and enriched for single cells based on forward scatter area and width (FSC-A x FSC-W). The mCherry+ cells were selected for further analysis, calculating the top and bottom 25% of BFP signal normalized to mCherry.
