# Supplementary material for: An mRNA processing pathway suppresses metastasis by governing translational control from the nucleus
Source: Nat Cell Biol. 2023 May 8;25(6):892–903. doi: 10.1038/s41556-023-01141-9 (PMC10264242; doi:10.1038/s41556-023-01141-9)
Supplement: Supplementary file 2 — Reporting Summary [file 41556_2023_1141_MOESM2_ESM.pdf]

nature portfolio

Corresponding author(s): Hani Goodarzi

Last updated by author(s): Mar 4, 2023

Reporting Summary

Nature Portfolio wishes to improve the reproducibility of the work that we publish. This form provides structure for consistency and transparency in reporting. For further information on Nature Portfolio policies, see our Editorial Policies and the Editorial Policy Checklist.

Statistics

For all statistical analyses, confirm that the following items are present in the figure legend, table legend, main text, or Methods section.

n/a	Confirmed	
☐	☒	The exact sample size (*n*) for each experimental group/condition, given as a discrete number and unit of measurement
☒	☐	A statement on whether measurements were taken from distinct samples or whether the same sample was measured repeatedly
☐	☒	The statistical test(s) used AND whether they are one- or two-sided<br>*Only common tests should be described solely by name; describe more complex techniques in the Methods section.*
☐	☒	A description of all covariates tested
☐	☒	A description of any assumptions or corrections, such as tests of normality and adjustment for multiple comparisons
☐	☒	A full description of the statistical parameters including central tendency (e.g. means) or other basic estimates (e.g. regression coefficient) AND variation (e.g. standard deviation) or associated estimates of uncertainty (e.g. confidence intervals)
☐	☒	For null hypothesis testing, the test statistic (e.g. *F*, *t*, *r*) with confidence intervals, effect sizes, degrees of freedom and *P* value noted<br>*Give P values as exact values whenever suitable.*
☒	☐	For Bayesian analysis, information on the choice of priors and Markov chain Monte Carlo settings
☒	☐	For hierarchical and complex designs, identification of the appropriate level for tests and full reporting of outcomes
☐	☒	Estimates of effect sizes (e.g. Cohen's *d*, Pearson's *r*), indicating how they were calculated

Our web collection on statistics for biologists contains articles on many of the points above.

Software and code

Policy information about availability of computer code

Data collection	No software was used for data collection.
Data analysis	Sequencing data was analyzed using custom R and Python scripts, using these tools: cutadapt (v2.3), FASTX-Toolkit (v0.0.13), umitools (v0.3.3), bowtie2 (v2.3.5), salmon (v0.14.1), ctk (v1.1.13), bwa (v0.7.17). The Ribolog and APAlog have been deposited to GitHub (https://github.com/goodarzilab/Ribolog, https://github.com/goodarzilab/APAlog). MaxQuant (v1.6.3.3) and Perseus (v1.6.2.3) were used for mass spectrometry data analysis. Living Image (v4.7.3) was used to acquire in vivo imaging data with IVIS instrument (Perkin Elmer).

For manuscripts utilizing custom algorithms or software that are central to the research but not yet described in published literature, software must be made available to editors and reviewers. We strongly encourage code deposition in a community repository (e.g. GitHub). See the Nature Portfolio guidelines for submitting code & software for further information.

Data

Policy information about availability of data

All manuscripts must include a data availability statement. This statement should provide the following information, where applicable:

- Accession codes, unique identifiers, or web links for publicly available datasets
- A description of any restrictions on data availability
- For clinical datasets or third party data, please ensure that the statement adheres to our policy

All sequencing data have been deposited in the GEO database under accession GSE186647. Proteomics data has been deposited in the PRIDE database under accession PXD029560. A previously published HNRNPC iCLIP dataset (E-MTAB-1371) was used in this study. The human breast cancer data were derived from the TCGA (at Genomic Data Commons, https://gdc.cancer.gov). The METABRIC data set was obtained from cBioPortal (https://www.cbioportal.org). The CPTAC breast

cancer data set was obtained from Proteomics Data Commons (https://proteomic.datacommons.cancer.gov/pdc/). All other data supporting the findings of this study are available from the corresponding author on reasonable request.

Field-specific reporting

Please select the one below that is the best fit for your research. If you are not sure, read the appropriate sections before making your selection.

☒ Life sciences ☐ Behavioural & social sciences ☐ Ecological, evolutionary & environmental sciences

For a reference copy of the document with all sections, see nature.com/documents/nr-reporting-summary-flat.pdf

Life sciences study design

All studies must disclose on these points even when the disclosure is negative.

Sample size	Based on our previous work (Goodarzi et al., Nature, 2014; Goodarzi et al., Cell, 2015; Goodarzi et al., Cell, 2016), for in vivo experiments, mice were distributed into cohorts with 4-5 mice per cohort, which in NSG background is enough to observe a >2-fold difference with 90% confidence. For example, at t=33 days, average normalized lung photon flux from colonized CN-LM1a breast cancer cells is 150 with s.d. of 66. Based on this distribution, with a cohort size of n=5 in each arm, we can detect a difference of ~100% in size with 90% power. Similarly, in MDA-LM2 cells in the same study, at t=33 days, average normalized signal was recorded as 291 and s.d. of 104, which suggests a cohort size of n=4.<br>For other experiments, no statistical methods were used to calculate sample size.
Data exclusions	No data were excluded from the analysis.
Replication	Cell migration/invasion assays were performed in 4 biological replicates. CoIP-MS, TMT-MS, RTqPCR, and western blot experiments were performed in biological triplicates. Sequencing-based experiments (Ribo-seq, RNA-seq, CLIP-seq, PAPERCLIP, MPRA) were performed in biological duplicates.
Randomization	Mice for in vivo experiments were randomly assigned into cohorts. For other experiments (molecular biology), no randomization was performed.
Blinding	For cell migration/invasion assays, the person counting the colonies was blinded for the experimental conditions. For other experiments, the data was acquired and analyzed by the same person and the blinding was not deemed necessary.

Reporting for specific materials, systems and methods

We require information from authors about some types of materials, experimental systems and methods used in many studies. Here, indicate whether each material, system or method listed is relevant to your study. If you are not sure if a list item applies to your research, read the appropriate section before selecting a response.

Materials & experimental systems

n/a	Involved in the study	
☐	☒	Antibodies
☐	☒	Eukaryotic cell lines
☒	☐	Palaeontology and archaeology
☐	☒	Animals and other organisms
☒	☐	Human research participants
☒	☐	Clinical data
☒	☐	Dual use research of concern

Methods

n/a	Involved in the study	
☒	☐	ChIP-seq
☐	☒	Flow cytometry
☒	☐	MRI-based neuroimaging

Antibodies

Antibodies used	anti-beta-tubulin (Proteintech 66240-1-Ig) (western blot loading control)<br>anti-GAPDH (Proteintech 60004-1-Ig) (western blot loading control)<br>anti-HNRNPC (Santa Cruz sc-32308) (western blot, IP, CLIP)<br>Mouse IgG (Jackson 015-000-003) (IP control)<br>anti-PABPC4 (Proteintech 14960-1-AP) (western blot, PAPERCLIP)<br>anti-TIA1 (Proteintech 12133-2-AP) (CLIP)<br>anti-ELAVL1 (Proteintech 11910-1-AP) (CLIP)<br>anti-PABPN1 (Proteintech 66807-1-Ig) (western blot)<br>anti-PDLIM5 (Proteintech 10530-1-AP) (western blot)<br>anti-TXNRD1 (Proteintech 11117-1-AP) (western blot)<br>anti-PGK1 (Proteintech 17811-1-AP) (western blot)<br>anti-FLAG tag (Proteintech 66008-1-Ig) (western blot)<br>anti-rabbit IgG, conformation specific, HRP (Cell Signaling 5127S) (western blot)

	anti-mouse IgG, Light chain specific, HRP (Cell Signaling 91196S) (western blot)<br>anti-rabbit IgG, IRDye 680RD (Li-Cor 926-68071) (western blot)<br>anti-mouse IgG, IRDye 800CW (Li-Cor 926-32210) (western blot)
Validation	All primary and secondary antibodies used in this study are commercially available and have been validated for used applications in human cells by the manufacturers. We have used recommended antibody dilutions for western blot experiments, and detected bands of expected molecular weight, as described by the manufacturers. Prior to CLIP-seq library preparation, the antibodies were tested in IP-western blot experiment, although the antibodies used were validated for IP by the manufacturers.

Eukaryotic cell lines

Policy information about cell lines

Cell line source(s)	Human cell lines used in this study are available from ATCC:<br>MDA-MB-231 (ATCC HTB-26)<br>HEK293T (ATCC CRL-3216)<br>HCC1806 (ATCC CRL-2335)<br>MDA-LM2 cell line, the lung metastatic derivative of MDA-MB-231, has been described (ref. 17 in the manuscript), and was a gift from Dr. Joan Massagué.<br>HCC1806-LM2c cell line, the lung metastatic derivative of HCC1806, has been described (Passarelli et al., Nat Cell Bio, 2022), and was a gift from Dr. Sohail Tavazoie.
Authentication	The cell lines were authenticated using STR profiling.
Mycoplasma contamination	All cell lines have been routinely tested for mycoplasma contamination by a qPCR based assay and tested negative.
Commonly misidentified lines<br>(See ICLAC register)	No commonly misidentified cell lines were used in this study.

Animals and other organisms

Policy information about studies involving animals; ARRIVE guidelines recommended for reporting animal research

Laboratory animals	Mice were housed in accordance with UCSF IACUC protocol in humidity- and temperature-controlled rooms on a 12 hour light-dark cycle with free access to food and water. Seven- to twelve-week-old age-matched female NOD scid gamma mice (NSG, Jackson Labs, 005557) were used in this study.
Wild animals	The study did not involve wild animals.
Field-collected samples	The study did not involve samples collected from the field.
Ethics oversight	This study complies with all relevant ethical regulations that are approved by UCSF Institutional Review Board (IRB) and Institutional Animal Care and Use Committee (IACUC, approval number AN179718).

Note that full information on the approval of the study protocol must also be provided in the manuscript.

Flow Cytometry

Plots

Confirm that:

- [x] The axis labels state the marker and fluorochrome used (e.g. CD4-FITC).
- [x] The axis scales are clearly visible. Include numbers along axes only for bottom left plot of group (a 'group' is an analysis of identical markers).
- [x] All plots are contour plots with outliers or pseudocolor plots.
- [x] A numerical value for number of cells or percentage (with statistics) is provided.

Methodology

Sample preparation	MDA-MB-231 cells were trypsinized, washed in 1x PBS, and resuspended in FACS buffer (1x PBS, 1% FBS, 1 mM EDTA) for analysis/sorting.
Instrument	The cells were sorted using FACSAria II (BD Biosciences) cell sorter, equipped with 360, 405, 488, 561 and 633 nm lasers.
Software	FACSDiva (BD Biosciences) was used to monitor data acquisition. FlowJo (BD Biosciences) was used for post-acquisition data analysis.
Cell population abundance	The cells were sorted based on the BFP/mCherry ratio, and 25% top and bottom fractions were collected for analysis. 300k (siCTRL-1), 350k (siCTRL-2), 400k (siHNRNPC-1) and 500k (siHNRNPC-2) cells per bin were sorted for RNA extraction. The purity of the cells post-sorting was not assessed.

Gating strategy

The cells were gated by FSC-A/SSC-A parameters, the single cells were enriched by FSC-A/FSC-W gating, the mCherry-positive cells were gated by comparing the library-expressing cells with untransduced MDA-MB-231 cells. In each sample, mCherry-positive cells were sorted based on the BFP/mCherry ratio into two bins, corresponding to 25% top and bottom fractions.

☒ Tick this box to confirm that a figure exemplifying the gating strategy is provided in the Supplementary Information.
