# Supplementary figures and images for: An mRNA processing pathway suppresses metastasis by governing translational control from the nucleus
Source: Nat Cell Biol. 2023 May 8;25(6):892–903. doi: 10.1038/s41556-023-01141-9 (PMC10264242; doi:10.1038/s41556-023-01141-9)

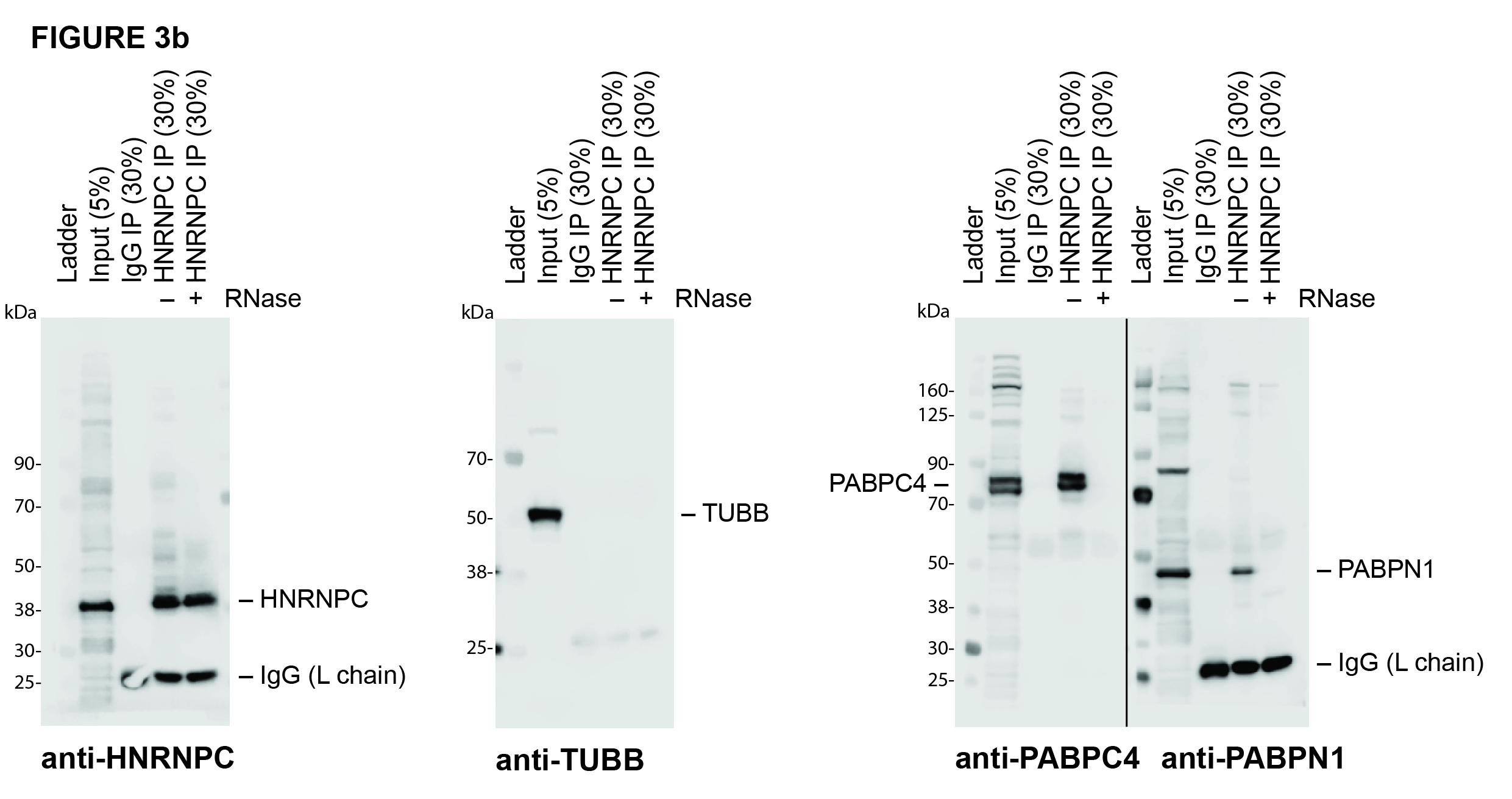

Supplement: Source Data Fig. 3 — Unprocessed blot images. [file 41556_2023_1141_MOESM5_ESM.jpg]

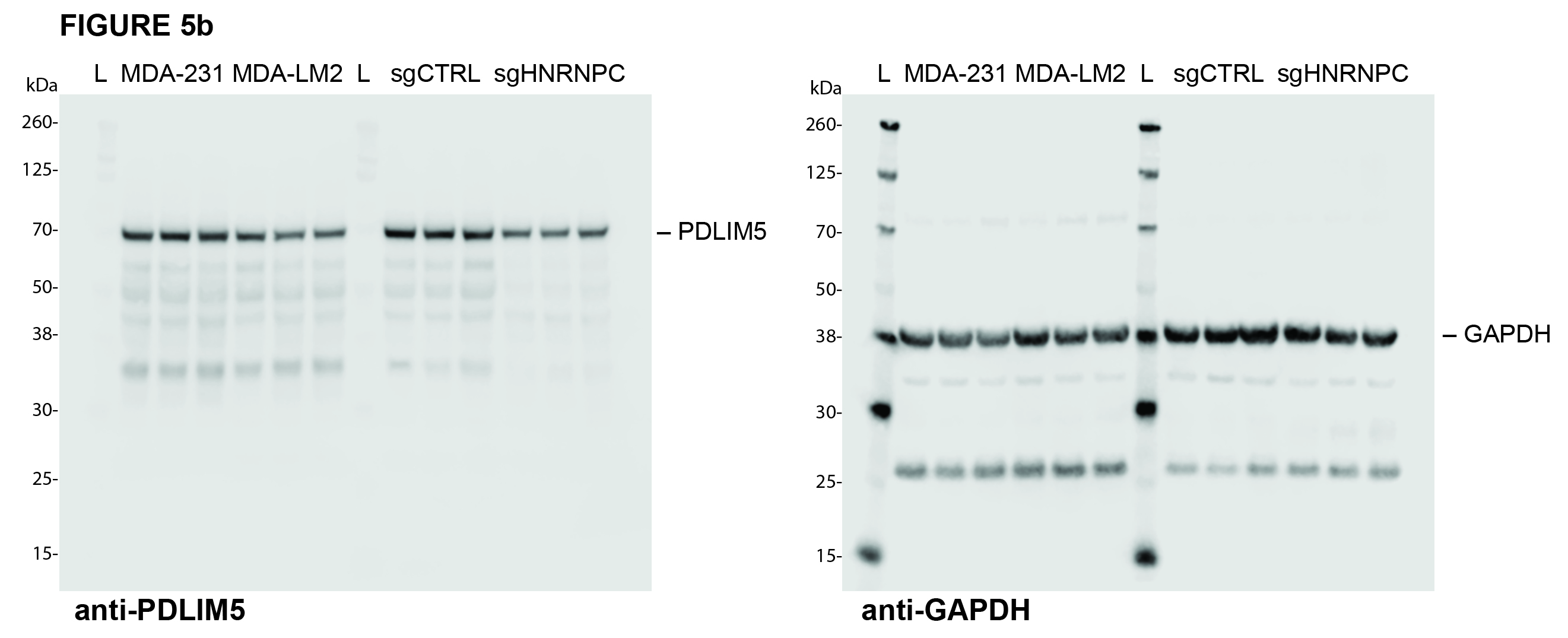

Supplement: Source Data Fig. 5 — Unprocessed blot images. [file 41556_2023_1141_MOESM8_ESM.jpg]

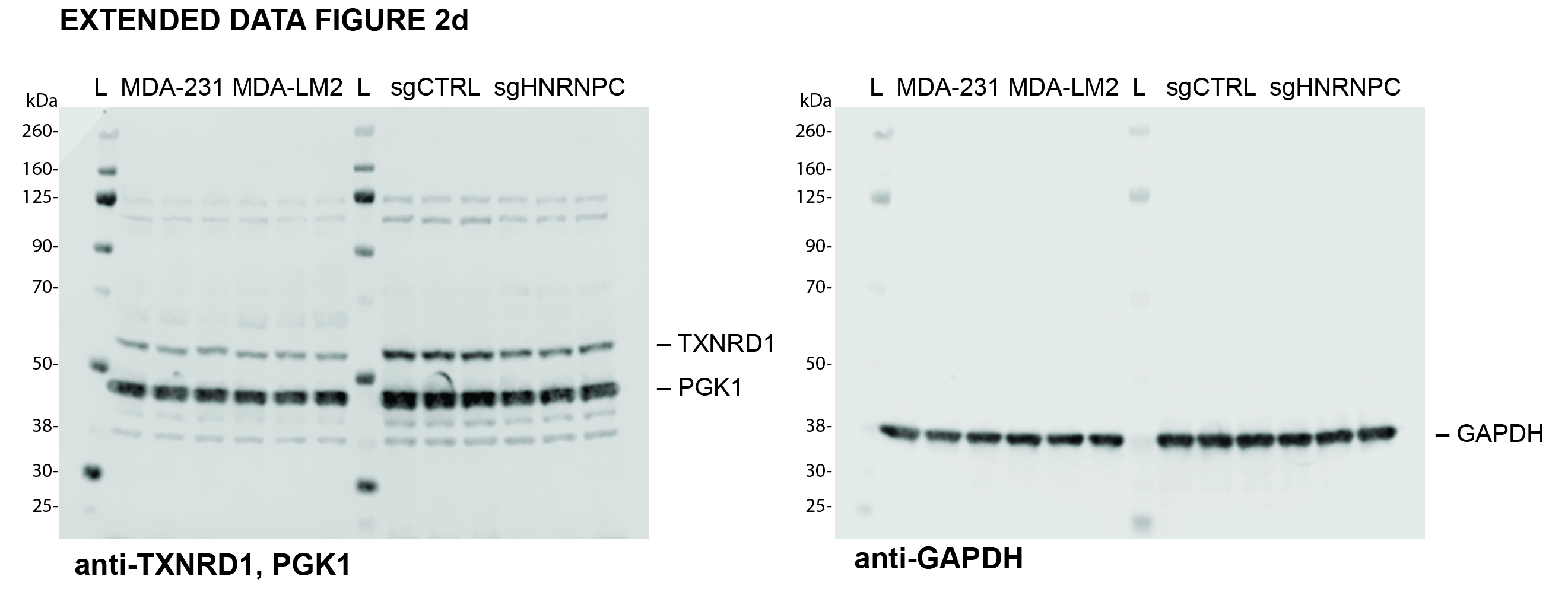

Supplement: Source Data Extended Data Fig. 2 — Unprocessed blot images. [file 41556_2023_1141_MOESM11_ESM.jpg]

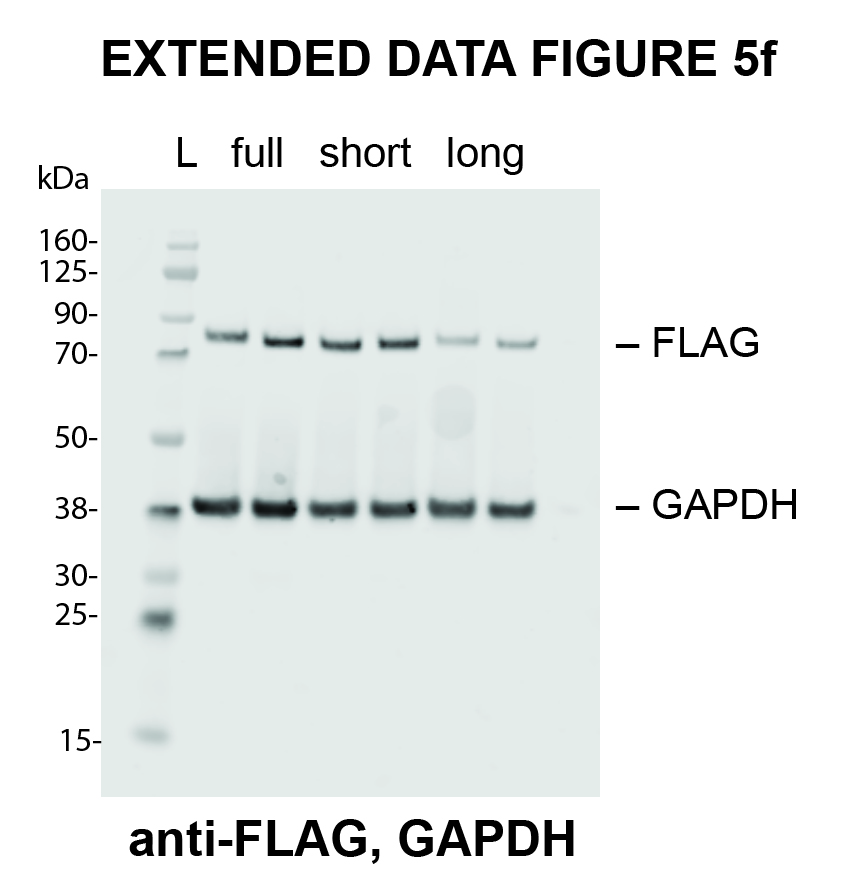

Supplement: Source Data Extended Data Fig. 5 — Unprocessed blot images. [file 41556_2023_1141_MOESM15_ESM.jpg]
